# Is Consumption Contagious?

**Published:** 1889-01-26

**Authors:** 


### Is Consumption Contagious?

Is consumption contagious? The general answer would be, "No." For generations we have been accustomed to look upon phthisis as an inevitably fatal but non-infectious disease. Perhaps both ideas are wrong; recent investigations seem to prove that the latter at least is erroneous. Perhaps in England we are too much used to consumption to note with care the method of its ravages; it is our national disease. "It was in the family," is held to be a sufficient explanation; or if there are absolutely no data to support this theory, if not even a great-aunt can be supposed to have died of it, the phrase is changed to, "It must have been in the family." And that is all that is said about it.

In face of the fact that in England one death out of every seven is still due to tubercular disease of the lungs, such indifference seems strange. If one death in seven occurred from leprosy or small-pox, what an outcry there would be! Is our placidity due to the fact that in consumption the disease attacks the inner tissue of the body, and its horrible nature is hidden from view. If we could realise the actual condition of the lungs of consumptives we would be more likely to ask ourselves if there is not at least a strong probability that anything affected by these diseased organs would convey infection to others. In America and Australia they are beginning to ask the question, and on the whole the answer is affirmative.

Various facts have been noted. It has been observed that health resorts for consumptives become at last chosen homes for the disease. One generation of invalid strangers after another comes and dies in them, and in the end the disease appears among the natives and becomes common. How does this happen? In many ways. The necessary intimacy of attendance on the dying, given all the more freely because no infection is feared, may lay the germs of the disease. It has been noticed that the laundresses employed in washing the clothes, especially the handkerchiefs, of consumptives, are apt to contract the disease, the probability of infection in their case being increased by their inhaling the poison in a hot, moist atmosphere, favourable to the development of the germs. It seems not unlikely that when two members of a family are attacked by consumption the old explanation of family taint may be thrown aside, a sufficient reason being that they were in the habit of sleeping together. Breathing the same air, the one that is first attacked—possibly from causes outside heredity—infects the other, and one life at least is needlessly sacrificed; while, unconscious that the sorrow might have been prevented, friends and relations murmur resignedly—

> "I have marked it well, it must be true,
> Death never takes one alone, but two."

Strangers, too, may be forced into unwilling companionship. Lately an Australian barrister, Dr. M'Mullen, protested in the *Australian Medical Gazette* against healthy and consumptive passengers being forced to share the same cabin in long voyages. The invalid probably objects to ventilation; the healthy man concedes the point, and suffers more or less from his courtesy. If he stands on his rights, every fit of coughing that attacks his companion will be set down to his account. This certainly ought to be prevented. Also, it may be suggested that the more or less inevitable promiscuity in which sailors sleep makes consumption such a common disease among them—hardy men who, one would think, would be the last to suffer from such a disease. This matter has been brought forward by a layman. It is well that it should be so; for unless they are supported by the common-sense of the community at large, all the efforts of all the doctors in the kingdom can do but little good.

